# Impact of financial literacy on household stock profit level in China

**DOI:** 10.1371/journal.pone.0296100

**Published:** 2023-12-18

**Authors:** Zhiyuan Luo, S. M. Ferdous Azam, Laixi Wang

**Affiliations:** 1 Graduate School of Management, Management and Science University, Shah Alam, Selangor, Malaysia; 2 School of Economics and Trade, Henan University of Technology, Zhengzhou, Henan, China; Yunnan Technology and Business University, CHINA

## Abstract

The popularization of financial literacy has become a global trend, with governments across the world expressing commitment to continuously enhancing the financial literacy of their citizens to improve the country’s overall financial well-being. However, there is a lack of research evaluating the actual effects of financial literacy on Chinese households. This study first investigated the micro impact of financial literacy on the household stock profit level using data from the 2019 China Household Finance Survey. As most existing studies use factor analysis to measure financial literacy from a single dimension of financial knowledge, our study additionally used the entropy method to construct a composite evaluation system of financial literacy from four dimensions: financial skills, knowledge, attitudes, and behaviors. The ordinary least squares model was utilized as the primary regression model to estimate the correlation, and the average financial literacy of other households in the same community was selected as an instrumental variable. Further instrumental variable regression analysis was conducted using the two-stage least squares method. Three robustness tests were performed to ensure the reliability of the research findings. The results demonstrate that financial literacy significantly enhances household stock profit levels. The mediation effect analysis indicates that financial literacy affects stock profit levels through financial information attention. Moreover, financial literacy has a more substantial promoting effect on stock profit levels for households with members working for state-owned enterprises and those living in first-tier cities. This study confirms the value of financial literacy; identifies important channels for residents to increase their property income; and provides important guidance for the government, educational organizations, and financial institutions. This also injects more vigor into market participation to improve the persistently sluggish Chinese stock market.

## 1. Introduction

Stock investment provides a channel for returns that are higher than inflation. Effective stock investment can increase household financial welfare. However, losses caused by poor investor decision-making also continue to occur. In 2015, the Shanghai and Shenzhen stock indexes experienced declines of over 40%, leading to an average per capita loss of approximately 510,000 yuan for investors. Data from five rounds of the Chinese Household Finance Survey conducted from 2011 to 2019 indicate that more than 50% of households experienced stock losses [1]. This poor decision-making is often owing to investors’ lack of financial literacy [2]. The global trend of promoting financial literacy gained momentum after the 2008 financial crisis. The Chinese authorities have consistently implemented initiatives aimed at improving the public’s financial literacy through organized literacy programs. The People’s Bank of China (PBOC) has established the bureau of financial consumer protection since 2012, which focuses on households’ financial education and financial rights protection, and has made national financial literacy and financial education a key priority. Over the past decade, Chinese households’ financial literacy has steadily increased and is above the global average [3]. However, the effect of implementing financial literacy remains a debatable issue.

Recent research suggests that financial literacy can improve returns on mutual fund investments [3]. Bianchi [4] noted that households with financial literacy adjust their investment portfolio’s risk exposure dynamically, leading to superior overall investment returns compared to families with lower financial literacy. However, being financially literate does not necessarily lead to investment returns. Instead, it reduces stock returns for less educated and elderly households [5]. Therefore, the research on the relationship between financial literacy and stock returns are subject to controversy. Furthermore, some financial information is relatively difficult for the public to access owing to the high cost of accessing information and limited attention. This will make it difficult for investors who lack financial literacy to discover and leverage potential trading information. Improved financial literacy contributes to the ability of investors to access, focus on, and analyze financial information [6]. In this context, it remains to be explored whether information attention is a potential mechanism by which financial literacy affects stock profit levels.

Therefore, this study aims to examine the effect of financial literacy on the stock profit level of Chinese households. We employ a direct measure of household welfare: the level of stock investment returns. While previous studies provided foundational insights, there remains a considerable scarcity in the analysis of differences in workplace ownership and city size within Chinese households. Such a research gap can be filled by exploring the heterogeneity in the influence of financial literacy on stock profitability levels. We also attempt to reveal information attention as a mechanism between financial literacy and stock market returns, providing a nuanced perspective for household investment decisions. Therefore, three specific questions are proposed in this study: “Does financial literacy impact stock profit levels?” “What is the heterogeneity of this effect?” and “Does financial literacy affect stock profit levels through attention to financial information?”

This study provides new empirical evidence for behavioral finance and investment decision-making theories, while also expanding the assessment of financial literacy implementation effects. Chinese households have experienced significant wealth growth through real estate investments over the past decade. Given the prevailing circumstances of China’s economic decline and the downturn in the real estate market, stock investment is an appropriate choice for diversified asset allocation. This provides important insights for guiding the importance of comprehensive financial literacy among households, encouraging the rational allocation of household wealth, and creating financial well-being. For policymakers, our study also provides an important basis for improving existing financial education policies from a multidimensional perspective. This can further ensure the quality of financial literacy and expand the community of beneficiaries.

We extract 13 relevant secondary indicators from the 2019 China Household Finance Survey data to investigate these research questions and construct a financial literacy index using the entropy method. In our empirical analysis, we employ an ordinary least squares (OLS) model and an instrumental variable regression analysis using the two-stage least squares method (IV-2SLS) for the primary regression analysis and endogeneity testing, respectively. Subsequently, we apply Probit and mediation effect models for robustness and mediation effect tests. Our findings reveal a significant positive impact of Chinese households’ financial literacy on stock profit levels. The regression analysis results of the three tests remain robust after replacing the dependent variable with stock profitability, excluding the samples working in the finance industry and risk aversion, and the independent variable with a financial literacy score. We argue that household financial literacy affects stock profit levels through attention to financial information. Owing to the heterogeneity of financial literacy and financial behavior, conducting heterogeneity analysis targeting different groups is more effective [7]. We find that for households with state-owned enterprise backgrounds and those living in first-tier cities, such an effect will be more pronounced.

Our study makes the following contributions. First, we construct a comprehensive evaluation system of financial literacy from four dimensions of financial knowledge, attitude, skills, and behaviors, which solves the imprecise measurement of financial literacy. Second, based on the effective measurement of financial literacy, this study evaluates the relationship between Chinese households’ financial literacy and stock profit level, enriching the research in this area. Furthermore, this study explores the mechanisms categorized as financial literacy, financial information attention, and the stock profit level channel. This improves the understanding of how financial literacy affects stock profit levels. Finally, this paper discusses the heterogeneous characteristics of financial literacy on stock profit levels in subgroups. This facilitates the formulation of targeted policies based on household characteristics and geographical differences to promote household property income.

The remaining sections of this paper are arranged as follows. Section 2 describes a literature review and hypotheses development. Section 3 presents the research methodology, including data sources, measurement of variables, model specification, and summary statistics. Section 4 reports the empirical findings, followed by further discussion in Section 5. Section 6 provides the conclusions, limitations, and policy recommendations.

## 2. Literature review and hypotheses development

### 2.1 Literature review

Considerable academic interest has developed around the factors influencing stock returns, such as regional financial development level [1], investor attention [8], investment risk and macroeconomics [9], asset growth [10], and investor sentiment [11]. Household investment decisions involve evaluating investment risks and returns, often requiring investors to leverage their intrinsic financial literacy. Financial literacy is a crucial determinant of individual investment decisions, as possessing a high level of financial knowledge enables better management and control of investment risks [5, 12].

The importance of financial literacy in household financial asset investment is growing, as it is a valuable kind of human capital. This is owing to the development of financial markets that require households to make more complex financial decisions than ever [13]. Financial literacy enhances investors’ stock market participation [14], increases household demand for risky financial assets [15], improves the identification of fraud in risky decision-making [16], and promotes financial inclusion [17]. Additionally, an individual’s financial literacy, experiences, attitudes, and behaviors can contribute to their financial decision-making [18]. However, the concept of financial literacy lacks a relatively precise definition. This is because financial literacy derives from multiple perspectives in different social and economic contexts, each of which has a unique scope. Most existing studies confuse financial literacy with financial knowledge. Financial knowledge is a fundamental comprehension that individuals ought to possess to effectively manage their finances [19]; and it is widely acknowledged as a fundamental element of financial literacy [7]. Nevertheless, Bongomin et al. [20] argued that individuals are financially literate when they demonstrate competence in making financial decisions and practically applying what they have learned. Therefore, financial knowledge and financial literacy are not the same. Financial skills empower individuals to make informed choices about their finances and reduce the likelihood of being deceived or misled. Lusardi [21] argued that there is a greater need for individuals to acquire financial skills. This is because individuals require a better understanding of various financial instruments, investment methods, and risk management strategies to navigate the increasingly complex financial landscape. Therefore, financial skills represent another crucial dimension of financial literacy. Additionally, individuals’ attitudes toward wealth can significantly influence their behavior toward achieving financial literacy and improving financial knowledge. For example, Strömbäck et al. [22] highlighted the profound impact of optimistic psychological traits on shaping individual financial behavior and subsequent well-being. Park and Sela [23] noted that individuals should strive to avoid financial decisions that are inconsistent with their emotional style. Therefore, financial behavior is seen as another important aspect of financial literacy.

Accompanied by good financial knowledge, skills, and attitudes, financial behavior is the ultimate step to making responsible and informed financial decisions. Clark et al. [24] believed that financial behavior plays a decisive role in the face of the vast array of product choices and financial information available in today’s market. This shows that there is no uniform academic consensus on the definition of financial literacy. To have a more accurate and comprehensive understanding of the meaning of financial literacy, several scholars, based on the collection and aggregation of relevant literature, have argued that financial literacy involves the knowledge, skills, attitudes, and behaviors necessary to achieve financial well-being [20]. The PBOC has also utilized these four dimensions to measure the overall financial literacy levels of the Chinese population [3].

### 2.2 Hypotheses development

According to modern portfolio theory, investors can optimize their stock investment portfolios with rich financial literacy and analytical ability. Kass-Hanna et al. [25] suggested that individuals with financial literacy tend to have positive financial behaviors, leading to the creation of social welfare. With improved financial literacy, family members gain a deeper understanding of financial markets, investment products, and economic factors that impact stock prices. This enables them to identify suitable investment opportunities and make informed choices aligned with their financial goals and risk tolerance. Households with higher levels of financial literacy tend to invest more efficiently and aggressively [26]. This also increases the participation and scale of household stock investment [27]. Bianchi [4] further identified that financial literacy significantly impacts portfolio performance, and households with higher levels of financial literacy are more likely to balance their portfolios to achieve higher returns. Increased financial literacy enhances their ability to diversify their investment portfolios effectively, spreading risks across different assets. They are more inclined to use diversified investment portfolios to avoid financial market risks, increasing stock investment returns. Moreover, investors with financial literacy can reduce the risk of loss, avoid overtrading and ignorance of investment fees, and thus achieve higher returns on mutual fund investments [3]. Hu and Huang [28] noted that financial literacy improves investors’ ability to select stocks and timing of trade, which positively contributes to financial returns. Therefore, we propose the following hypothesis.

**H1:** Financial literacy is positively related to stock profit level.

Owing to the continuous development of information technology, the research perspective on financial information has been increasingly emphasized. Xu et al. [26] noted that information attention is an indispensable mediating mechanism between financial knowledge and financial asset allocation performance. Household investors must collect and pay attention to trading information in their buying and selling decisions. Thus, there may be transmission paths of financial literacy, information attention, and investment profit level. Theoretically, the transmission of financial transaction information in household investment is incomplete, and household investors cannot pay full attention to all valuable information in their investment decisions. Thus, information asymmetry is a barrier to household stock profits. However, financial literacy tends to improve the efficiency with which households pay attention to and process information. Higher levels of financial literacy imply that household investors obtain and focus on valuable trading information through various channels, filtering out irrelevant information. Choi and Choi [6] argued that investors with higher levels of financial literacy could actively search for and discuss economic and financial information, such as financial reports and economic surveys, which dynamically affect the outcome of household financial asset allocation performance. Gargano and Rossi [29] suggested that investors’ attention to information is correlated with better investment performance. When investors acquire valuable trading information, their transactions become more profitable. As financial literacy not only directly influences stock profitability but also indirectly influences it through financial information attention, we propose the following hypothesis:

**H2:** Financial information attention mediates the relationship between financial literacy and stock profit level.

Owing to the widespread existence of individual differences within households, the impact of financial literacy on stock profit levels could vary. Yi and Zhang [30] noted that China’s first-tier cities play a leading and radiation-driven role in regional economic activities. City size, as an important socio-economic factor, affects households’ access to financial literacy and the speed of information acquisition. The efficacy of asset allocation optimization is higher among households in economically developed cities in China with higher financial literacy [27]. These phenomena can be ascribed to the accessibility advantage enjoyed by households located in first-tier cities, which grants them quicker access to a diverse array of financial information and investment opportunities. Contrastingly, small- and medium-sized cities often face scarce financial resources, which is further compounded by a relatively low level of financial awareness among households. In addition, first-tier cities are generally the financial centers of the region, with better financial infrastructure and services, and the overall financial climate has an inherent advantage, allowing residents to have more access to high-quality financial resources.

Additionally, differences, such as in workplace ownership, could also generate heterogeneous effects. According to the classification of sample households’ work units, they can be divided into state-owned and private enterprises. State-owned enterprises (SOEs) are usually directly owned or controlled by the government, with a relatively centralized decision-making system and a stable management level. The objectives of SOEs are related to social responsibility and political interests, such as stabilizing prices, promoting employment, and increasing the value of assets. Contrastingly, private enterprises are usually owned by private individuals, partners, or shareholders. Their decision-making mechanisms are relatively flexible and more susceptible to market competition and profitability pressures [31]. Because of this contextual difference, SOEs and private firms differ significantly in terms of employee training, promotion opportunities, and compensation systems [32]. Employees working in SOEs could have easier access to first-hand financial knowledge and educational resources because SOEs can provide a more stable work environment, training opportunities, and relevant career development plans. However, owing to fierce market competition, the private sector could prioritize business-related training rather than individual financial literacy improvement. Therefore, differences in aspects such as workplace ownership could cause financial literacy to have differentiated impacts on stock profit levels. Based on the above analysis, we propose the following research hypotheses:

**H3:** Workplace ownership heterogeneity exists in the relationship between financial literacy and stock profit level.
**H4:** City size heterogeneity exists in the relationship between financial literacy and stock profit level.

## 3. Materials and methods

### 3.1 Data source

The data used in our research are from the China Household Financial Survey (CHFS). The 2019 data are the latest accessible official data as of today. Data were accessed for research purposes since March 16, 2022. The authors do not possess any information that can potentially reveal the identities of the individual participants during or after the process of data collection. The CHFS uses a stratified, three-stage, and probability-proportional-to-size sampling method, with the sample covering 29 provinces in China. The sample frame is divided into three sampling stages according to the level of administrative districts at an urban level, neighborhood and village committees, and community households. Each stage of sampling uses population size as the weighting factor. To ensure randomness and representativeness, CHFS utilizes its self-developed vector map to determine the random starting point and calculate the sampling intervals, ultimately obtaining a valid household financial sample. The CHFS database was obtained after seeking official permission and authorization without violating ethical guidelines. Our study diligently followed the standard reporting guidelines of the CHFS. The Ethics Committee of the Management and Science University exempted this study from ethics approval and waived the requirement for informed consent as the study used existing data from CHFS (exemption no. EA-L1-01-GSM-2023-09-0002). All relevant data are within the paper and its supporting information (S1 Data).

### 3.2 Variables measurement

The dependent variable of interest in this study is household stock profit level. Most existing studies use the income from stock sales minus the input from stock buying to measure stock profit and loss [28, 33]. However, the fluctuation of stock book prices does not represent the return generated from actual trading. Therefore, this study is based on the questionnaire distributed by CHFS (2019) on stock asset returns that asked respondents: “How much profit or loss did your household generate from stock trading last year?” and “How much income did your household receive from stock dividends last year?” The household stock profitability level is calculated by summing the stock gains and dividends.

Determining the conceptual dimensions of financial literacy is an essential prerequisite for conducting measurements. Huston [34] compiled relevant literature on financial literacy measurements and identified four main conceptual dimensions of financial literacy: financial skills, financial knowledge, financial attitudes, and financial behaviors. Some studies have also shown that household financial literacy is determined by financial knowledge, attitudes, skills, and behaviors [35, 36]. Additionally, Huston [34] argued that a comprehensive measurement of financial literacy based on the four dimensions mentioned above can ensure the objectivity and comprehensiveness of financial literacy assessment. Combining the above views, this study constructs a comprehensive evaluation system of financial literacy from four dimensions: financial knowledge, financial attitudes, financial skills, and financial behaviors.

Huston [34] argued that the generally accepted measurement rule in developing a standardized financial literacy indicator system should include at least 2–5 items to assess the target dimension. Four dimensions require at least 8–20 items to ensure the accuracy of the measurement. Accordingly, 13 secondary indicators are selected from the CHFS (2019), as shown in Table 1. Among them, financial knowledge includes five questions on the calculation of simple interest, compound interest, inflation, stock risk identification, and fund risk identification. We assign a value of 1 to the financial knowledge with correct answers and 0 to the rest of the response options. Financial behavior included whether to use credit cards, participate in the financial market, and engage in financial borrowing and lending behaviors. Financial attitudes involve attitudes toward stock investment and project investment. Financial skills are reflected in retirement insurance plans, financial budget management, and household asset-liability management. We assign a value of 1 to items that meet the above descriptions of financial behavior, attitudes, and skills and a value of 0 otherwise.

**Table 1:** Financial literacy evaluation system.

Primary indicator	Secondary indicator	China Household Financial Survey Project items	Option assignment
Financial knowledge	Simple interest calculation	Assuming the bank’s interest rate is 4% per annum, what is the principal and interest at maturity if 100 RMB is deposited for a one-year fixed term?	The correct option is assigned as 1, while the incorrect option is assigned as 0.
	Compound interest calculation	What is the total principal and interest after two years on 10,000 RMB compounded at 10% per annum?	
	Inflation calculation	How would the purchasing power of a 100 RMB deposit in a bank change after one year, given an annual inflation rate of 8% and a bank deposit interest rate of 5%?	
	Stock risk identification	Which carries higher risk: main board stocks or start-up board stocks?	
	Fund risk identification	Which carries higher risk: equity funds or bond funds?	
Financial behavior	Credit card usage	Have you used a credit card?	The response option “Yes” equals 1, “No” equals 0.
	Participation in financial markets	Have you purchased the following financial products: financial wealth management, stocks, funds, bonds, non-RMB assets, and derivatives?	
	Financial lending	Have you borrowed or lent money?	
Financial attitudes	Return-risk matching	How do you evaluate the risk and return of stocks?	High-risk and high return option equals 1, while others are equal to 0.
	Risk preference	If you have a sum of money for investment, which type of investment project would you prefer to choose?	Option unknown and unwilling to take any risks equal to 0, while other options equal to 1.
Financial skills	Retirement insurance plan	Have you participated in social pension insurance?	The response option “Yes” equals 1, and “No” equals 0.
	Financial budget management	Is your household income greater than your total consumption?	
	Asset and liability management	Are your household assets greater than your total liabilities?	

After completing the measurement of the secondary indicators, our study further obtained aggregated financial literacy. Most current studies use factor analysis to reduce the dimensionality of the original information for assessing financial literacy [27, 37, 38]. Moreover, for evaluation systems with more indicators, researchers assign values to responses to different questions and calculate scores by summation [28]. As the number of secondary indicators for evaluating financial literacy is not equal, subjectively setting the weight of indicators can cause estimation bias. Based on Chen’s [39] method, the weight of each indicator is determined by calculating the entropy value of the indicator to achieve an objective evaluation of financial literacy. The main advantage of this method is that it can reflect the effect value of the indicators’ information entropy and avoid the bias caused by subjective factors. Its calculation is based on the degree of variability of each indicator value to determine the corresponding weight. Therefore, comparing the subjective assignment method has higher accuracy and credibility.

Our research adapts control variables proposed by previous studies [28, 33] and mediation variables suggested by [26]. For measurement purposes, data for each household are normalized. Table 2 presents how our study collects the control and mediation variables’ data.

**Table 2:** Control and mediation variable measurement.

Variables	Measurement description
Age	2019 minus the year of birth of the household member interviewed
Sex	The dummy variable is defined as 1 for male and 0 for female
Higher education	The dummy variable is defined as 1 for bachelor’s degree and above and 0 for other
Financial information attention	Ordinal variable is defined as 1 = not concerned at all, 2 = a little concerned, 3 = generally concerned, 4 = very concerned, 5 = extremely concerned
Marriage	The dummy variable is defined as 1 for married and 0 for other
Total asset	Household total asset
Household size	Number of people in the household

Source: Developed by the researcher

### 3.3 Model specification

As both the independent and dependent variables are continuous, this study first uses an OLS linear regression model to test the effect of financial literacy on stock profit levels. The benchmark Model (1) is set up as follows:

$${\mathrm{Profit}\_\mathrm{level}}_{i}{=\alpha +\beta }_{1}{\mathrm{Financial}\_\mathrm{literacy}}_{i}{+\beta }_{2}{\mathrm{Control}}_{i}{+\varepsilon }_{i}$$ (1)

Here, I = 1, 2, 3, …, N, represents the individual household. As described in the Variables Measurement section, profit level denotes the summation of the stock gains and dividends. Financial literacy stands for the level of financial literacy estimated by the entropy method. Control represents all the control variables, ε is a random disturbance that satisfies the standard normal distribution assumption, and α is constant. All the β are the estimates of the parameters.

The dependent variable, stock profitability, conducted in the robustness test is a binary discrete variable conforming to (0~1) the probability density function distribution. Therefore, we use the Probit model to test the impact. The Model (2) is set up as follows:

$${\mathrm{Profitability}}_{i}{=1(\alpha +\beta }_{3}{\mathrm{Financial}\_\mathrm{literacy}}_{i}{+\beta }_{4}{\mathrm{Control}}_{i}{+\mu }_{i}>0)$$ (2)

Here, μ ~ (0, σ^2^), profitability is a dummy variable. When a household makes a profit, it equals 1, otherwise equals 0. The rest of the explanation is the same as above.

There is a possible mediating effect if the independent variable can directly affect the dependent variable and indirectly affect the dependent variable through other channels [40, 41]. Based on the above research hypothesis that financial information attention could mediate between financial literacy and stock profit level, this study establishes a mediating effect model to test these relationships. The financial information attention is an ordered variable that must be analyzed by establishing the Ordered-Probit model, and the specific model process is set as follows:

$${\mathrm{Information}\_\mathrm{attention}}_{i}{=\alpha +\beta }_{5}{\mathrm{Financial}\_\mathrm{literacy}}_{i}{+\varepsilon }_{i}$$ (3)

$${\mathrm{Profit}\_\mathrm{level}}_{i}{=\alpha +\beta }_{6}{\mathrm{Financial}\_\mathrm{literacy}}_{i}{+\beta }_{7}{\mathrm{Information}\_\mathrm{attention}}_{i}{+\beta }_{8}{\mathrm{Control}}_{i}{+\varepsilon }_{i}$$ (4)

Here, information attention denotes the potential mediating variable. The rest of the explanations remain the same as described above. First, the mediated effects model tests the effect of financial literacy on stock profit level using the same model as in Model (1). Second, it tests whether the effect of financial literacy on the mediating variable of financial information attention is significant by using Model (3). Finally, financial literacy and information attention are simultaneously used as independent variables to construct a regression, Model (4), for stock profit.

### 3.4 Summary statistics

In data processing, the initial step involves extracting and retaining relevant variables from the original CHFS (2019) dataset pertinent to this study. Subsequently, the three datasets are merged based on the household identification code, and missing samples are removed. Subsequently, each household financial literacy index is constructed using the entropy method based on the scores of the secondary indicators. Further, to mitigate the impact of outliers, we take the natural logarithm of total assets and stock profit level of each sample household after adding 1. Consequently, 15,961 valid samples were obtained.

The descriptive statistics give the sample characteristics and distribution. Table 3 reports the scores of the secondary financial literacy indicators for Chinese households. The average financial knowledge and skills scores are higher than those for financial attitude and behavior. Within financial knowledge, the highest score is observed for understanding inflation, while the lowest score is for fund risk identification. Regarding financial behavior, credit card usage receives a higher score than participation in financial markets and financial borrowing. Regarding financial attitude, the highest score is attributed to risk preference. Within financial skills, household assets and liability management scores surpass those for retirement insurance planning and financial budget management.

**Table 3:** Descriptive statistics of scores on secondary indicators of financial literacy.

Primary indicator	Score range	Secondary indicator	Score	Frequency	Percentage	Average score
Objective financial knowledge	0–5	Simple interest calculation	0	10,974	68.76	0.31
			1	4,987	31.24	
		Compound interest calculation	0	11,743	73.57	0.26
			1	4,218	26.43	
		Inflation calculation	0	8,373	52.46	0.47
			1	7,588	47.54	
		Stock risk identification	0	13,651	85.53	0.14
			1	2,310	14.47	
		Fund risk identification	0	14,439	90.46	0.095
			1	1,522	9.54	
Financial behavior	0–3	Credit card usage	0	11,783	73.82	0.26
			1	4,178	26.18	
		Participation in financial markets	0	13,091	82.02	0.18
			1	2,870	17.98	
		Financial lending	0	15,833	99.20	0.008
			1	128	0.80	
Financial attitudes	0–2	Return-risk matching	0	12,898	80.81	0.19
			1	3,063	19.19	
		Risk preference	0	8,445	52.91	0.47
			1	7,516	47.09	
Financial skills	0–3	Retirement insurance plan	0	2,471	15.48	0.84
			1	13,490	84.52	
		Financial budget management	0	7,056	44.21	0.56
			1	8,905	55.79	
		Asset and liability management	0	196	1.23	0.99
			1	15,765	98.77	

Source: Developed by the researcher

Table 4 reports descriptive statistics for the variables. The financial literacy of Chinese households ranges from 0 to 1, with an average of 0.19 above the median of 0.15. The secondary indicator of financial literacy has the largest mean value for financial knowledge, followed by financial skills. Financial attitudes are slightly higher than financial behaviors. The mean value of household stock profit level is 1690 yuan. The mean level of household financial information attention is 1.99, corresponding to little attention. The average age of heads of household in the sample is 53 years, ranging from 18 to 96 years; 72% of household heads are men; and married households account for 86%. The proportion of households with higher education is 13%. The average household size is 3.11 people. The average total assets of households amount to 1,620,000 yuan, which is approximately $221,756.

**Table 4:** Summary statistics.

Variables		N	Mean	SD	Min	Max
Independent Variables	Financial literacy	15961	0.19	0.14	0	1
	Financial knowledge	15961	0.073	0.085	0	0.401
	Financial behavior	15961	0.029	0.047	0	0.371
	Financial attitude	15961	0.033	0.040	0	0.136
	Financial skills	15961	0.056	0.023	0	0.091
Dependent Variable	Stock return (Unit: 10000RMB)	15961	0.169	1.71	0	100
Mediating Variable	Information attention	15961	1.99	1.02	1	5
Control Variables	Age	15961	52.99	14.08	18	96
	Sex	15961	0.72	0.45	0	1
	Household size	15961	3.11	1.43	0	15
	Marriage	15961	0.86	0.35	0	1
	Higher education	15961	0.13	0.34	0	1
	Total asset (unit: 10000 RMB)	15961	162	264	0.025	11900

Source: Developed by the researcher

## 4. Results

To test the research hypotheses, this study first uses the OLS model to establish baseline linear regression analysis and the IV-2SLS method to examine the endogeneity of the model. In addition, three robustness tests are used to verify the consistency and reliability of the research findings. Further, the mediation effect model is used to test the financial literacy mechanism on stock profit levels. Finally, a subsample analysis is conducted to assess the heterogeneous effect of financial literacy on stock profit levels.

### 4.1 Benchmark regression analysis

Table 5 reports the baseline regression analysis results of financial literacy on the household stock profit level, in which Column (1) presents the results without control variables, while Column (2) includes regression analysis results with control variables for the age of the household member interviewed, sex, household size, marital status, higher education, and total household assets. As evident from Columns (1) and (2) of Table 5, financial literacy significantly positively affects household stock profit (p < .01). Thus, hypothesis H1 is supported, indicating a notable enhancement in stock profitability owing to improved financial literacy among Chinese households. The estimated coefficients of financial literacy in Columns (1) and (2) of Table 5 also reveal that a one-unit increase in financial literacy is associated with a respective increase of 5.3134 and 5.0407 units in stock profit level. For the control variables, an increase in the age of the household results in the household possessing and accumulating more financial literacy, which significantly increases the stock profit. This is consistent with Zhang et al.’s [33] finding. However, households with male heads are weaker than those with female heads regarding stock profit levels. The larger the household size, the more likely it is to reduce the level of household stock profit. This is because an increase in the size of the household diverts a lot of time and energy from household members [42].

**Table 5:** Financial literacy and stock profit level.

Variables	Stock profit level			
	OLS	OLS	OLS (First stage)	IV-2SLS (Second stage)
	(1)	(2)	(3)	(4)
Financial literacy	5.3134***	5.0407***		7.1734***
	(0.1917)	(0.1970)		(0.3519)
IV Financial Literacy			0.7346***	
			(0.0164)	
Age		0.0091***	-0.0013***	0.0121***
		(0.0010)	(0.0000)	(0.0012)
Sex		-0.0616*	0.0094***	-0.0621*
		-0.0616*	(0.0022)	(0.0350)
Household size		-0.0246***	-0.0004	-0.0111
		(0.0092)	(0.0007)	(0.0117)
Marriage		0.0990**	-0.0004	0.1048**
		(0.0453)	(0.0030)	(0.0479)
Higher education		0.1274*	0.0547***	-0.0465
		(0.0675)	(0.0031)	(0.0558)
Total asset		0.0859***	0.0195***	0.0143
		(0.0105)	(0.0007)	(0.0160)
Observations	15,961	15,961	15,961	15,961
R^2^	0.1413	0.1500	0.2996	0.1321
F			1997.3	
Wu–Hausman (p)			42.188	
			0.001	

Note

***p < .01

**p < .05

*p < .1

Moreover, larger family sizes necessitate a higher budget allocation for daily needs, which often constrains the amount available for investment stock. Further, marriage can significantly affect the level of household stock profit, mainly owing to married households having wider social networks and diversified sources of income [28]. This enables them to access more information on stock trading and pursue high-yield stock investment opportunities, which is supported by our findings. Regarding total household assets and education, the family’s accumulated wealth and higher education positively correlate with stock profit levels. This is consistent with the findings of Merkoulova and Veld [43].

### 4.2 Endogeneity: Instrumental variable two-stage least squares

Considering the potential endogeneity in the benchmark model, household investors will focus on nurturing financial literacy after earning investment returns, leading to a possible reverse causality between stock returns and financial literacy. Further, the entropy-based approach employed for measuring financial literacy has strong explanatory power, but inherent measurement errors are inevitable. Moreover, the omitted variables could lead to either an overestimation or underestimation of the effect of financial literacy. To mitigate the abovementioned endogeneity, this study adopts an instrumental variable approach. Following Lusardi and Mitchell’s [44] method, the average financial literacy level of the surveyed households’ communities (excluding the surveyed household itself) is chosen as the instrumental variable. The instrument’s validity is contingent upon fulfilling the first assumption of relevance, in which household respondents become more financially literate when exposed to higher financial literacy in the same community. Further, the instrument satisfies the second exogeneity assumption. The financial literacy of others is beyond the respondent’s control and does not directly impact their level of stock profitability. Consequently, employing the average financial literacy level within the surveyed households’ communities as an instrument variable is appropriate.

Table 5 of Columns (3) and (4) presents the results of the instrumental variable analysis. The findings of the first-stage regression analysis are presented in Column (3). They indicate a substantial and positive correlation between the instrumental variable and financial literacy (p < .01). Simultaneously, in the instrumental variable test, the first-stage F-value of 1997.3 is much greater than 16.38. Foo et al. [45] suggested that the F-value exceeds the critical value of 16.38 (p < .1), indicating that the average financial literacy within the same communities is not a weak instrumental variable. Further, the p-value of the Hausman test is highly significant, which rejects the null hypothesis that there is no endogeneity in the model, indicating that financial literacy is endogenous. As shown in Column (4) of Table 5, after correcting for endogeneity concerns, the coefficient of financial literacy remains significantly positive (p < .01). With a one-unit increase in financial literacy, stock investment returns increased by 7.1734 units. This proves that improved financial literacy significantly and positively affects stock profit levels. A comparative analysis of the coefficients of financial literacy in Column (4) against those in Columns (1) and (2) of Table 5 reveals that adopting the IV-2SLS estimation, which eliminates endogeneity, yields markedly higher coefficient estimates for financial literacy. This underscores that financial literacy is a pivotal determinant of household stock investment returns, and overlooking endogeneity could result in underestimating its effect.

### 4.3 Robustness test

To eliminate the possible estimation bias introduced by the entropy approach, three robustness tests were conducted to support the validity of the research findings. Table 6 reports the results of the three robustness tests. First, since households working in the financial sector could have higher financial literacy and some conservative households are risk-averse, such households may prefer risk-free investment options. Attempts have been undertaken to improve the precision and applicability of the research outcomes in our study. Column (1) of Table 6 excludes samples working in the financial sector and samples whose investment preferences entail risk aversion. The coefficient in Column (1) of Table 6 shows that financial literacy positively affects stock profit. This implies that the relationship between financial literacy and stock profit remains significant (p < .01), even after these samples are eliminated. Further, an alternative approach involves substituting the independent variable with an aggregated score of 13 secondary indicators. Since all 13 secondary indicators are binary variables, a value of 1 is assigned when the binary variable equaled 1, allowing the calculation of scores for each secondary indicator as indicated in the descriptive statistics in Table 3. Consequently, we aggregate the scores of these secondary indicators and employ the total financial literacy score as an independent variable to conduct OLS regression analysis on stock profit level, as shown in Column (2). After the substitution, financial literacy continues to significantly affect stock profit levels (p < .01), with an estimated effect of 0.2894. Additionally, the stock profit level is substituted with household stock profitability. The CHFS (2019) asks, “What are the actual profit and loss earned from stock trading in the last year?” Dummy variables are used to indicate household stock profitability. A value of 1 represents households that made a profit on stocks last year, while 0 indicates households that either broke even or made a loss. As the dependent variable is a dummy variable, the Probit model is used for regression analysis in Column (3) of Table 6. The average marginal effects of financial literacy remain significantly positive, even in this altered specification (p < .01). The results of all three robustness tests show that financial literacy can significantly and positively affect stock profits, indicating that the results of the empirical analyses are robust.

**Table 6:** Robustness test.

Variables	Stock profit level		Stock profitability
	OLS	OLS	Probit
	(1)	(2)	(3)
Financial literacy	5.9271***		0.0434***
	(0.2818)		(0.0048)
Financial literacy (aggregate scores)		0.2894***	
		(0.0111)	
Controls	Yes	Yes	Yes
Observations	7352	15961	15961
R^2^	0.1591	0.1347	0.2297

Notes: (1) Parameters in Columns (1) and (2) are estimated t-values; (2) Parameters in Column (3) are average marginal effects; (3)

***p < .01

**p < .05

*p < .1

### 4.4 Mediation effect test

The research hypotheses explain that financial literacy can impact stock profit levels by enhancing household financial information attention. Our study further tests this mechanism using a mediated effects model, and Table 7 reports the findings. The benchmark regression analysis in Column (1) of Table 7 indicates that financial literacy is positively related to stock profit level (p < .01), consistent with the results of the benchmark regression analysis. Columns (2) and (3) of Table 7 present the results of the mediation effect test of financial information attention. The coefficient of financial literacy on information attention is 3.5141 and is significant (p < .01), suggesting that financial literacy significantly impacts households’ attention to financial information. From Column (3) of Table 7, after adding the mediating variable of information attention, the coefficient of the effect of financial literacy on the level of stock profit is 4.6293, which is lower than that of 5.0407 in Column (1) of Table 7. This suggests a partial mediating effect, and Hypothesis H2 is supported. Further, the Sobel test also reveals that the mediating effect of financial information attention accounts for 8.16% of the total effect.

**Table 7:** Mediation effect of information attention.

Variables	Profit level	Information attention	Profit level
	OLS	Ordered Probit	OLS
	(1)	(2)	(3)
Financial literacy	5.0407***	3.5141***	4.6293***
	(0.1970)	(0.0697)	(0.2009)
Information attention			0.1334***
			(0.0185)
Controls	Yes	Yes	Yes
Sobel test			7.93
			(0.000)
Proportion of total effect mediated			8.16%
Observations	15,961	15,961	15,961
R^2^	0.1500	0.1026	0.1535

Note

***p < .01, **p < .05, *p < .1

### 4.5 Heterogeneity analysis

There is considerable variation in the degree of financial literacy among various types of business ownership and city sizes. To further explore these heterogeneities, this study employs sub-group tests. Table 8 presents the results of this analysis, in which Columns (1) and (2), respectively, show the regression analysis outcomes of financial literacy’s impact on stock profit level for households employed in SOEs and private enterprises. The findings indicate that the estimations of the subsample in both scenarios demonstrate a significant positive correlation. The coefficient of financial literacy in Column (1) exhibits more significance compared to that in Column (2). This suggests that the impact of improved financial literacy on stock profitability is more prominent for households with members employed in state-owned firms as opposed to those employed in private enterprises. Therefore, Hypothesis H3 is confirmed. The possible explanation is that households with SOEs usually have more stable employment relationships and income sources, and employees are relatively less exposed to the risk of unemployment and salary fluctuations. This makes it easier for them to have a stable source of funds to invest in stocks, and they are more likely to hold stocks for a long time to earn more profits. Additionally, household members who work in SOEs could be exposed to more specialized financial literacy training, which also gives them an incentive to engage in stock investment.

**Table 8:** Heterogeneity test.

Variables	Stock profit level			
	State-owned enterprise		First-tier city	
	Yes	No	Yes	No
	OLS	OLS	OLS	OLS
	(1)	(2)	(3)	(4)
Financial literacy	6.5290***	4.5235***	6.2334***	3.8604***
	(0.4207)	(0.2224)	(0.3127)	(0.2501)
Control variables	Yes	Yes	Yes	Yes
Observations	2,974	12, 987	5,941	10020
R^2^	0.1867	0.1328	0.1598	0.1193

Note: ***p < .01, **p < .05, *p < .1

The results of the heterogeneity test of financial literacy on stock profit levels for households residing in first-tier and non-first-tier cities are presented in Columns (3) and (4) of Table 8. The findings suggest that there is a considerable positive relationship between financial literacy and stock profit levels, irrespective of the household’s location in terms of city size. The impact coefficient of financial literacy in first-tier cities is 6.2334, higher than that of non-first-tier cities at 3.8604. This finding suggests that the influence of financial literacy on the profitability of household stocks is more pronounced in first-tier cities than in non-first-tier cities. Therefore, hypothesis H4 is supported. A possible explanation for the above results is that first-tier cities usually have more sophisticated financial infrastructure and resources, making it easier for households to access financial information and investment opportunities. This contributes to enhancing households’ financial literacy, thereby increasing their awareness of both potential dangers and opportunities associated with investments. Consequently, it enables them to make informed and sensible decisions while engaging in stock market activities.

## 5. Discussion

### 5.1 Financial literacy helps improve stock returns

The growing importance of financial literacy is gaining prominence with the increasing interconnectedness of global economies and information accessibility. Although previous studies examined many effects of financial literacy [14–17], there is still a dearth of scholarly investigation of the correlation between financial literacy and stock returns, particularly within the Chinese stock market context. This study addresses the aforementioned research gap by uncovering a positive correlation between the prevalence of financial literacy and micro-household stock returns. This partly confirms Jiang et al.’s [3] study that financial literacy improves returns on mutual funds in China. Our findings support the continuous promotion of financial literacy activities as a means to empower individual investors as well as foster a more resilient and responsive stock market. Our analyses further uncover the varied effects of these impacts across different city tiers and ownership enterprises. The influence of financial literacy on stock profitability is notably stronger for households living in first-tier cities and working in SOEs as compared to their counterparts. This highlights the importance of implementing long-term strategies to ensure the sustainability of financial literacy programs, which provide a broader economic policy to mitigate the effects of market heterogeneity and reduce the incidence of suboptimal investment choices, thereby promoting more stable stock returns.

### 5.2 Financial information attention is an important mechanism by which financial literacy improves stock profit

Stock return levels can be better understood by considering the impact of financial literacy on households’ attention to financial information. As the level of financial literacy among households rises, the degree of attention that household investors devote to financial information also increases. This heightened focus on financial information is associated with higher stock returns. This differs from Hu and Huang’s [28] information inhibition theory and is largely consistent with Xu et al.’s [26] information facilitation theory. The latter argues that financial literacy improves, and residents with a greater number of information channels can obtain more information conducive to their own investment decisions. They can also summarize and compare information obtained from different channels, which is conducive to enhancing investment returns. A knowledgeable and information-sensitive investor base improves market efficiency, which increases stock returns and enhances overall market performance. Our findings highlight the importance of financial literacy in facilitating the relationship between investor information sensitivity and stock returns and advocate the integration of effective financial education initiatives with information attention mechanisms to optimize investment outcomes.

## 6. Conclusions

Based on the 2019 CHFS data, this study uses the entropy value method to construct a comprehensive evaluation system of financial literacy from four dimensions—financial knowledge, financial skills, financial attitudes, and financial behaviors—and further empirically examine the relationship between financial literacy and Chinese households’ stock profit level. The findings indicate a strong and positive correlation between financial literacy and the level of household stock profits. The above research finding remains valid and robust using endogeneity and robustness tests. Further, mediation effects analysis shows that financial information attention can explain part of the effect of financial literacy on stock profit levels. Specifically, financial literacy enhances households’ attention to economic and financial information, promoting households’ access to financial information and thus enhancing stock profitability. The heterogeneity analysis reveals that financial literacy promotes the level of stock profit more strongly among households working in SOEs and those living in first-tier cities as compared to their counterparts.

Considering these research findings, it is crucial to formulate concrete policy recommendations to promote financial literacy among Chinese households. One effective approach could involve implementing financial education programs targeting different demographic groups, tailored to their specific needs and levels of financial knowledge. These programs require a greater focus on cultivating household financial attitudes, behaviors, and skills alongside financial knowledge. Furthermore, collaboration between the government, financial institutions, and educational organizations is essential to develop and disseminate these educational initiatives widely. Interactive workshops, online courses, and community-based seminars could be organized to reach a broader audience. Incorporating financial literacy education into the school curriculum at various levels can help instill financial awareness from an early age. Additionally, it is imperative to expand the channels for acquiring and disseminating financial information, thereby enhancing residents’ attention to effective information. Traditional media and digital platforms could be used to make the latest economic and financial information easily accessed in a timely manner. This approach aims to increase investors’ focus on financial information and improve decision-making efficiency. To maximize the impact of these initiatives, it is crucial to monitor and evaluate their effectiveness regularly. Surveys and assessments can be conducted to measure the improvement in financial literacy levels among the target population. Based on these evaluations, the programs can be refined and adjusted to ensure their relevance and effectiveness. Moreover, financial institutions can play a pivotal role by offering user-friendly financial tools and resources, accompanied by educational materials, to empower individuals to make informed financial decisions. These resources should be available both online and offline to meet the needs of individuals with differing levels of financial literacy. In summary, the successful implementation of these recommendations requires a coordinated effort among government agencies, financial institutions, educational organizations, and community stakeholders. By investing in financial education and empowering individuals with the necessary knowledge and skills, China can foster a financially literate population, leading to improved financial well-being and economic stability for the nation.

Although this study revealed some important findings, there are also some limitations. The entropy method calculates the financial literacy index based on the information entropy values and their corresponding weights of the indicators. However, it could overlook the interconnections between secondary indicators. For instance, there might be certain connections between four dimensions of financial literacy that the entropy method might fail to capture. Additionally, the entropy method does not involve the selection of indicators during the calculation process, unlike factor analysis and principal component analysis, which identify the indicators contributing the most to the variance. To this end, one endogeneity and three robustness tests were conducted to support the reliability of the research findings. Furthermore, the most effective solution would be to conduct a questionnaire survey to obtain up-to-date primary data and further construct a second-order structural equation model to assess these impacts. Four first-order latent variables can reflect the second-order financial literacy, and the structural equation model can capture the relationships between each first-order latent variable, enabling a more accurate estimation process.

## Supporting information

S1 Data (XLSX)

## References

[1] Zhang Y, Lu X. Regional financial development and stock earnings: evidence from the survey data of individual investors. J Yunnan Univ Fin Econ. 2021;37: 46–62. doi: 10.16537/j.cnki.jynufe.000727

[2] Kawamura T, Mori T, Motonishi T, Ogawa K. Is financial literacy dangerous? Financial literacy, behavioral factors, and financial choices of households. J Jpn Int Econ. 2021;60: 101131. doi: 10.1016/j.jjie.2021.101131

[3] Jiang J, Liao L, Wang Z, Xiang H. Financial literacy and retail investors’ financial welfare: evidence from mutual fund investment outcomes in China. Pac Basin Fin J. 2020;59: 101242. doi: 10.1016/j.pacfin.2019.101242

[4] Bianchi M. Financial literacy and portfolio dynamics. J Fin. 2018;73: 831–859. doi: 10.1111/jofi.12605

[5] Li J, Li Q, Wei X. Financial literacy, household portfolio choice and investment return. Pac Basin Fin J. 2020;62: 101370. doi: 10.1016/j.pacfin.2020.101370

[6] Choi S, Choi WY. Effects of limited attention on investors’ trading behavior: evidence from online ranking data. Pac Basin Fin J. 2019;56: 273–289. doi: 10.1016/j.pacfin.2019.06.007

[7] Lusardi A, Mitchell OS. The economic importance of financial literacy: theory and evidence. Am Econ J J Econ Lit. 2014;52: 5–44. doi: 10.1257/jel.52.1.5 28579637 PMC5450829

[8] Chen J, Tang G, Yao J, Zhou G. Investor attention and stock returns. J Financ Quant Anal. 2022;57: 455–484. doi: 10.1017/S0022109021000090

[9] Widagdo B, Jihadi M, Bachitar Y, Safitri OE, Singh SK. Financial ratio, macro economy, and investment risk on Sharia Stock Return. J Asian Fin Econ Bus. 2020;7: 919–926. doi: 10.13106/jafeb.2020.vol7.no12.919

[10] Artikis PG, Diamantopoulou L, Papanastasopoulos GA, Sorros JN. Asset growth and stock returns in European equity markets: implications of investment and accounting distortions. J Corp Fin. 2022;73: 102193. doi: 10.1016/j.jcorpfin.2022.102193

[11] Lv Y, Piao J, Li B, Yang M. Does online investor sentiment impact stock returns? Evidence from the Chinese stock market. Appl Econ Lett. 2022;29: 1434–1438. doi: 10.1080/13504851.2021.1937490

[12] Cupák A, Fessler P, Hsu JW, Paradowski PR. Investor confidence and high financial literacy jointly shape investments in risky assets. Econ Modell. 2022;116: 106033. doi: 10.1016/j.econmod.2022.106033

[13] Bannier CE, Schwarz M. Gender- and education-related effects of financial literacy and confidence on financial wealth. J Econ Psychol. 2018;67: 66–86. doi: 10.1016/j.joep.2018.05.005

[14] Fong JH, Koh BSK, Mitchell OS, Rohwedder S. Financial literacy and financial decision-making at older ages. Pac Basin Fin J. 2021;65: 101481. doi: 10.1016/j.pacfin.2020.101481

[15] Zhao T, Chen K, Wang Q, Luo C. Financial literacy, liquidity constraints and household risk asset allocation. Fin Res Lett. 2023;58: 104555. doi: 10.1016/j.frl.2023.104555

[16] Du W, Chen M. Too much or less? The effect of financial literacy on resident fraud victimization. Comput Hum Behav. 2023;148: 107914. doi: 10.1016/j.chb.2023.107914

[17] Zahid RMA, Rafique S, Khurshid M, Khan W, Ullah I. Do women’s financial literacy accelerate financial inclusion? Evidence from Pakistan. J Knowl Econ. 2023: 1–23. doi: 10.1007/s13132-023-01272-2

[18] Khurshid M, Zahid RMA, Nisa MU. Factors affecting financial decisions of university students: evidence from Pakistan. Manag Fin. 2023. doi: 10.1108/MF-05-2021-0207

[19] Chang D, Chen W, Tai X, Si Y. The impact of financial literacy on rural household self-employment: the mediating role of financial ability. Emerg Mark Fin Trade. 2022;58: 3297–3308. doi: 10.1080/1540496X.2022.2043152

[20] Okello Candiya Bongomin G, Mpeera Ntayi J, Akol Malinga C. Analyzing the relationship between financial literacy and financial inclusion by microfinance banks in developing countries: social network theoretical approach. Int J Sociol Soc Pol. 2020;40: 1257–1277. doi: 10.1108/IJSSP-12-2019-0262

[21] Lusardi A. Financial literacy and the need for financial education: evidence and implications. Swiss J Econ Stat. 2019;155: 1–8. doi: 10.1186/s41937-019-0027-5

[22] Strömbäck C, Lind T, Skagerlund K, Västfjäll D, Tinghög G. Does self-control predict financial behavior and financial well-being? J Behav Exp Fin. 2017;14: 30–38. doi: 10.1016/j.jbef.2017.04.002

[23] Park JJ, Sela A. Not my type: why affective decision makers are reluctant to make financial decisions. J Consum Res. 2018;45: 298–319. doi: 10.1093/jcr/ucx122

[24] Clark R, Lusardi A, Mitchell OS. Financial knowledge and 401(k) investment performance: a case study. J Pension Econ Finan. 2017;16: 324–347. doi: 10.1017/S1474747215000384

[25] Kass-Hanna J, Lyons AC, Liu F. Building financial resilience through financial and digital literacy in South Asia and Sub-Saharan Africa. Emerg Mark Rev. 2022;51: 100846. doi: 10.1016/j.ememar.2021.100846

[26] Xu S, Yang Z, Ali ST, Li Y, Cui J. Does financial literacy affect household financial behavior? The role of limited attention. Front Psychol. 2022;13: 906153. doi: 10.3389/fpsyg.2022.906153 35795410 PMC9252460

[27] Lu X, Xiao J, Wu Y. Financial literacy and household asset allocation: evidence from micro-data in China. J Consum Aff. 2021;55: 1464–1488. doi: 10.1111/joca.12406

[28] Hu S, Huang T. Financial literacy, information channels, and household investment returns. Invest Res. 2021;3: 20–32.

[29] Gargano A, Rossi AG. Does it pay to pay attention? Rev Financ Stud. 2018;31: 4595–4649. doi: 10.1093/rfs/hhy050

[30] Yi P, Li W, Zhang D. Sustainability assessment and key factors identification of first-tier cities in China. J Clean Prod. 2021;281: 125369. doi: 10.1016/j.jclepro.2020.125369

[31] He X, Wang C, Yang X, Lai Z. Do enterprise ownership structures affect financial performance in China’s power and gas industries? Util Policy. 2021;73: 101303. doi: 10.1016/j.jup.2021.101303

[32] Li M, Lien JW, Zheng J. Optimal subsidies in the competition between private and state-owned enterprises. Int Rev Econ Fin. 2021;76: 1235–1244. doi: 10.1016/j.iref.2019.11.011

[33] Zhang Y, Lu X, Xiao JJ. Does financial education help to improve the return on stock investment? Evidence from China. Pac Basin Fin J. 2023;78: 101940. doi: 10.1016/j.pacfin.2023.101940

[34] Huston SJ. Measuring financial literacy. J Con Aff. 2010;44: 296–316. doi: 10.1111/j.1745-6606.2010.01170.x

[35] Potrich ACG, Vieira KM, Kirch G. Determinants of financial literacy: analysis of the influence of socioeconomic and demographic variables. Rev Contab Finanç. 2015;26: 362–377. doi: 10.1590/1808-057x201501040

[36] Singh C, Kumar R. Financial literacy among women–Indian scenario. Univ J Account Fin. 2017;5: 46–53. doi: 10.13189/ujaf.2017.050202

[37] Sevim N, Temizel F, Sayılır Ö. The effects of financial literacy on the borrowing behaviour of Turkish financial consumers. Int J Con Stud. 2012;36: 573–579. doi: 10.1111/j.1470-6431.2012.01123.x

[38] Murendo C, Mutsonziwa K. Financial literacy and savings decisions by adult financial consumers in Zimbabwe. Int J Consumer Stud. 2017;41: 95–103. doi: 10.1111/ijcs.12318

[39] Chen P. Effects of normalization on the entropy-based TOPSIS method. Expert Syst Appl. 2019;136: 33–41. doi: 10.1016/j.eswa.2019.06.035

[40] Iacobucci D. Mediation analysis and categorical variables: the final frontier. J Consum Psychol. 2012;22: 582–594. doi: 10.1016/j.jcps.2012.03.006 PMC3501728 23180961

[41] Wen Z, Fang J, Xie J, Ouyang J. Methodological research on mediation effects in China’s mainland. Adv Psychol Sci. 2022;30: 1692. doi: 10.3724/SP.J.1042.2022.01692

[42] Lu X, Guo J, Gan L. International comparison of household asset allocation: micro-evidence from cross-country comparisons. Emerg Mark Rev. 2020;43: 100691. doi: 10.1016/j.ememar.2020.100691

[43] Merkoulova Y, Veld C. Stock return ignorance. J Financ Econ. 2022;144: 864–884. doi: 10.1016/j.jfineco.2021.06.016

[44] Lusardi A, Mitchell OS. Financial literacy around the world: an overview. J Pension Econ Financ. 2011;10: 497–508. doi: 10.1017/S1474747211000448 28553190 PMC5445931

[45] Foo JK, Doan T. The impact of sleep quality on mental health in working Australians: a quasi-experimental approach. Soc Sci Med. 2023;329: 116039. doi: 10.1016/j.socscimed.2023.116039 37379637

